# Tocotrienols for normalisation of hepatic echogenic response in nonalcoholic fatty liver: a randomised placebo-controlled clinical trial

**DOI:** 10.1186/1475-2891-12-166

**Published:** 2013-12-27

**Authors:** Enrico Magosso, Mukhtar Alam Ansari, Yogheswaran Gopalan, Ibrahim Lutfi Shuaib, Jia-Woei Wong, Nurzalina Abdul Karim Khan, Mohamed Rizal Abu Bakar, Bee-Hong Ng, Kah-Hay Yuen

**Affiliations:** 1 Advanced Medical and Dental Institute, Universiti Sains Malaysia, Kepala Batas, Malaysia; 2 School of Pharmaceutical Sciences, Universiti Sains Malaysia, Penang, Malaysia; 3 Faculty of Pharmacy, Universiti Teknologi MARA, Puncak Alam, Malaysia; 4 Hovid Research Sdn Bhd, Penang, Malaysia

## Abstract

**Background:**

Nonalcoholic fatty liver disease (NAFLD) is one of the commonest liver disorders. Obesity, insulin resistance, lipid peroxidation and oxidative stress have been identified amongst the possible hits leading to the onset and progression of this disease. Nutritional evaluation of NAFLD patients showed a lower-than-recommended intake of vitamin E. Vitamin E is a family of 8 isoforms, 4 tocopherols and 4 tocotrienols. Alpha-tocopherol has been widely investigated in liver diseases, whereas no previous clinical trial has investigated tocotrienols for NAFLD. Aim of the study was to determine the effects of mixed tocotrienols, in normalising the hepatic echogenic response in hypercholesterolaemic patients with ultrasound-proven NAFLD.

**Methods:**

Eighty-seven untreated hypercholesterolaemic adults with ultrasound-proven NAFLD were enrolled and randomised into control group (*n* = 44) and tocotrienols group (*n* = 43). The treatment, either mixed tocotrienols 200 mg twice daily or placebo, had a 1-year duration.

Normalisation of hepatic echogenic response, being the trial primary aim, was used in sample size calculations. The data were assessed according to intention to treat principle as primary outcome. Per protocol analysis was also carried out as secondary outcome measurement.

**Results:**

Thirty and 34 participants concluded the study in the tocotrienols and placebo group respectively. Alpha-tocopherol levels were within the normal range for all subjects. As primary outcome, the normalisation of hepatic echogenic response was significantly higher for the tocotrienols treated group compared to the placebo group in the intention to treat analysis (*P* = 0.039; 95% CI = 0.896-6.488). As secondary objective, the per protocol assessment also showed significant rate of remission (*P* = 0.014; 95% CI = 1.117-9.456). Worsening of NAFLD grade was recorded in two patients in the placebo group, but none in the group treated with tocotrienols. No adverse events were reported for both groups.

**Conclusion:**

This is the first clinical trial that showed the hepatoprotective effects of mixed palm tocotrienols in hypercholesterolemic adults with NAFLD.

**Trial registration:**

Clinicaltrials.gov, NCT00753532.

## Background

In recent years research on non-alcoholic fatty liver disease (NAFLD) has been intensified indicating an increased interest in this common hepatic disorder. Lipid peroxidation and oxidative stress have been recognised as playing a pivotal role in the pathogenesis and progression of NAFLD [1,2]. Nutritional assessment of NAFLD patients showed a lower-than-recommended intake of vitamin E [3], possibly leading to low plasma concentration of antioxidants. Recent clinical studies suggested vitamin E to be one of the most promising agent for amelioration of non-alcoholic steatohepatitis (NASH) in adults [4] and up to a certain extent in youngsters [5]. The most common homologue of the vitamin E family investigated is alpha-tocopherol. Other homologues of vitamin E, namely tocotrienols, also exist in isomers designated as alpha, beta, gamma and delta, and they differentiate from analogous isomers of tocopherol by the presence of an unsaturated phytyl chain (‘tail’) as opposed to a saturated one in tocopherols.

Tocotrienols are preferentially distributed to the liver [6,7] and alpha-tocotrienol has been reported to be 40-60 times more potent than alpha-tocopherol against lipid peroxidation in rat liver microsomes [8].

Prevalence of NAFLD in Asia has been showed in a recent review to be similar to Western countries [9], with local data in hypercholesterolaemic adults indicating a NAFLD prevalence of about 60% [10]. Concerns about the high prevalence of NAFLD and the not fully understood reasons for its progression to more severe form of liver diseases, have led to the initiation of this interventional, double-blind placebo-controlled study. The aim of the investigation was to evaluate the activity of tocotrienols in normalising hepatic echogenic response in adults with NAFLD with hypercholesterolaemia as an associated risk factor.

## Methods

### Patients

Volunteers of both genders aged 35 years and above with mild untreated hypercholesterolaemia and ultrasound-proven NAFLD, selected from a population-based study on the prevalence of NAFLD [10] were assessed for eligibility. Mild hypercholesterolemia, assessed during screening, was defined as ranging between 5.2 and 6.2 mmol/L (200-240 mg/dL) for total cholesterol and between 2.6 mmol/L and 4.2 mmol/L (100-161 mg/dL) for LDL-cholesterol. Alcohol intake, assessed through interview, had to be less than 20 g/day. Alanine transaminase (ALT), aspartate transaminase (AST) and gamma-glutamyl transpeptidase (GGT) had to be below 3 times the respective upper limit value of 53 IU/L, 40 IU/L and 49 IU/L for male or 32 IU/L for female.

Volunteers were excluded if they were presented in the 3 months prior to enrolment with anti-hyperlipidemic treatment and/or vitamin E intake and history of abuse or excessive intake of alcohol, previous cardiovascular events or hepatitis. No specific dietary guidance was given to the participants, however they were advised about the overall health benefits of increased physical activity and fat-reduced diet.

Participants were recruited at Universiti Sains Malaysia facilities in Kepala Batas Hospital (Penang, Malaysia), between February 2008 and June 2009. Out of 102 hypercholesterolaemic adults with ultrasound-proven NAFLD assessed for eligibility, 87 fulfilled the inclusion criteria and were enrolled by the investigators EM and YG, who also dispensed the treatments.

The present interventional study (http://www.clinicaltrials.gov/show/NCT00753532), designed as a parallel double-blind placebo-controlled study, was approved by the Research Ethics Committee for Human Studies of Universiti Sains Malaysia. Participants were enrolled upon signing informed consent.

### Randomisation and treatment

The treatment assigned to participants for 1 year was either mixed tocotrienols 200 mg twice daily or placebo. The content in each capsule of the mixed tocotrienols preparation was 61.5 mg, 112.8 mg and 25.7 mg for alpha-, gamma- and delta-tocotrienol, respectively and 61.1 mg of alpha-tocopherol. Both placebo and tocotrienols, were soft gel capsules and were similar in terms of colour, size, shape and surface texture. Participants were randomised using a computer generated random allocation sequence. A permuted block design was employed. Each block of specified size contained allocation ratio of 1:1 (placebo:tocotrienols). The random allocation sequence would select the next block and determine the next allocations. Sequence was not made known to researchers who enrolled participants. For the purpose of assigning participants to interventions, a subject number was used. The researcher (WJW) who generated the random allocation sequence and assigned participants was blinded to subjects’ clinical data and was independent from the persons who enrolled participants.

The mixed tocotrienols preparation (Tocovid Suprabio®) and the placebo capsules were purchased from Hovid (Ipoh, Malaysia). Researchers and volunteers were blinded to the assigned treatment.

### Clinical and metabolical evaluation

Quarterly, after an overnight fast, a 12 ml blood sample from each volunteer was withdrawn and analysed by an accredited laboratory for levels of serum total cholesterol (TC), LDL-cholesterol, HDL-cholesterol, triglycerides (TG), apolipoprotein B (ApoB), lipoprotein A (LP(A)), high-sensitivity C-reactive protein (hs-CRP), fasting glucose, serum creatinine, alkaline phosphatase (ALP), ALT, AST and GGT. Body mass index (BMI) and blood pressure were measured. Moreover, participants were asked to recall their lifestyle habits (such as food intake and physical activity). Compliance to the study treatment was assessed through patient recall, capsule count and plasma levels of tocotrienols. Plasma tocotrienol concentrations were measured using a validated high-performance liquid chromatographic (HPLC) method [11] after completion of the study, until then the samples were kept at -80°C. Quantitative HPLC analysis of plasma tocotrienol concentrations were performed to determine adherence to the treatment. Investigation of the association of plasma level of tocotrienols with response was not the aim of the present study.

High sensitivity B-mode ultrasonography (USG) examination was performed at baseline and after 1 year by the same experienced radiologists (MAA and ILS, in presence of a third radiologist, MRAB) using the same instrument, a Pentax-Hitachi EUB6500 (Tokyo, Japan) fitted with a EUP-C516 (3.5-5.0 MHz) probe using the standard adult abdominal settings, throughout the study. The radiologists were unaware of the clinical, metabolic and USG baseline conditions of the subjects. The USG evaluation consisted of a visual scoring system evaluating three aspects of interest: hepatorenal echodiscrepancy, posterior echo-penetration and portal vein wall clarity. Each of the three hepatic aspects considered was given a score of zero, if normal, 1 or 2 according to the echogenicity of the response [12]. Hepatic aspects and relative scoring system are detailed in Table 1. The values were then summed up and a total score of 3 and above was diagnosed as fatty liver. Steatosis was graded as mild (score 3), moderate (score 4) or severe (score 5-6). Only diffuse hyperechogenicity of the liver parenchyma was acceptable, whereas focal or patchy responses were excluded.

**Table 1:** Criteria of the hepatic aspects considered and relative scoring system for ultrasonographic diagnosis of nonalcoholic fatty liver disease

***Criteria***	***Score***
**Liver/kidney parenchyma**	
a) Homogeneous echotexture and absence of significant contrast with kidney parenchyma	0
b) Slight increase in liver/kidney echodiscrepancy	1
c) Extreme echodiscrepancy between liver and kidney	2
**Portal vein walls clarity**	
a) Clear definition of portal vein walls and structures	0
b) Decreased definition of portal vein walls and structures	1
c) Blurred visualisation of portal vein walls	2
**Posterior echo-penetration**	
a) Clear definition of hepatic structures from diaphragm	0
b) Decreased definition of liver and diaphragm structures	1
c) Blurred diaphragm with loss of definition	2

(Adapted from [12]).

### Statistical analysis

Prospective estimation of the sample size for a binary outcome trial with 5% significance level (alpha) and 80% power was calculated according to Pocock [13]. Therefore, the minimum number of patients required per arm was calculated to be 20. The binary outcomes considered were normalisation of echogenic response *versus* no-improvement or worsening.

Difference in proportion of participants that have been found with normal echogenic response at conclusion of the study between each group of treatment were assessed using Helmert-Pearson’s Chi-square test. The effects of independent variables, such as blood parameters and anthropometric data, were evaluated using ANOVA for split-plot design adjusted for unweighted-means [14]. Homogeneity of baseline characteristics was calculated using Wilcoxon Rank Test. Statistical calculations were performed using Numbers’09 (Apple, Cupertino, CA, USA) and QuickCalcs or InStat 3.1a for Macintosh (GraphPad Software, San Diego California USA).

The study primary end-point was a normalisation of hepatic echogenic response, defined as homogeneous hepatic echotexture with absence of significant contrast with kidney parenchyma, clear definition of portal vein walls and clear definition of hepatic structures from diaphragm (Table 1). The data were assessed according to intention to treat and per protocol analyses. Drop-out patients, as well as those with worsened condition, were computed as non-responders.

## Results

### Patients characteristics

**Figure 1:**
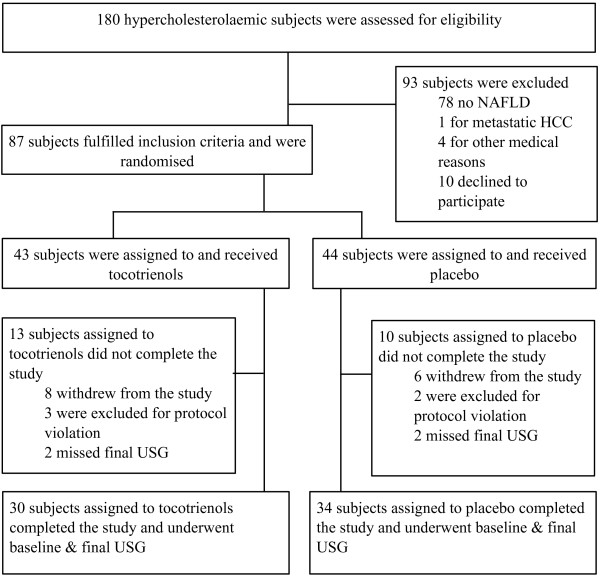
**CONSORT Flow-chart of the clinical trial, with reason for exclusion of assessed subjects and for subjects that did not concluded the study.** HCC = Hepatocellular carcinoma; USG = ultrasonography examination.

The study participants self-reported mostly sedentary lifestyles, with none of them engaged in regular physical exercise of minimum 30 minutes 3-time weekly. Out of 87 enrolled patients 64 completed the investigation. In the tocotrienols group 8 subjects withdrew consent, 3 were excluded for protocol violation and 2 did not come for the final USG follow-up. Similarly, in the placebo group 6 subjects withdrew consent, 2 were excluded for protocol violation and 2 did not come for the final USG follow-up. Initiating anti-hyperlipidemic therapy was the protocol violation that led to the exclusion of 5 participants from the study. Flowchart of the study is shown in Figure 1.

Overall, mean baseline characteristics of the 87 enrolled subjects were aged 51 ± 8 years (ranging from 36 to 74 years), BMI 27.2 ± 4.6 kg/m^2^, TC 5.7 ± 0.6 mmol/L, AST and ALT 37 ± 13 IU/L and 37 ± 19 IU/L, respectively. Thirty subjects (34.5%) were normoweight (BMI below 25), 37 (42.5%) overweight (BMI ranging between 25 and 30), 19 (21.8%) obese (BMI between 30 and 40) and only 1 (1.1%) subject was morbidly obese (BMI between 40 and 50). Nine subjects (10.3%) had impaired fasting glucose (IFG) above 7.0 mmol/L. Mild fatty liver was the most common finding with 69 (79.3%) cases, while moderate and severe fatty liver were found in 15 (17.2%) and 3 (3.4%) subjects, respectively. Ten participants (11.5%) were smokers, equally distributed amongst tocotrienols (11.6%) and placebo groups (11.4%), respectively. The two groups of enrolled subjects were found to be homogeneous with regard to baseline characteristics (Table 2).

**Table 2:** Details of all subjects enrolled

**Variable**	**Tocotrienols (n = 43)**	**Placebo (n = 44)**	***P *value**
Gender (female)	23 (53.5%)	30 (68.2%)	0.160
Age in years (range)	52 ± 9 (36-74)	49 ± 7 (38-68)	0.177
BMI (kg/m^2^)	27.2 ± 4.6	27.1 ± 4.6	0.873
Normoweight (BMI < 25)	16 (37.2%)	14 (31.8%)	
Overweight (25 < BMI < 30)	16 (37.2%)	21 (47.7%)	
Obese (30 < BMI < 40)	11 (25.6%)	8 (18.2%)	
Morbidly Obese (40 < BMI < 50)	0 (0.0%)	1 (2.3%)	
TC (mmol/L)	5.9 ± 0.6	5.6 ± 0.6	0.075
HDL (mmol/L)	1.44 ± 0.35	1.37 ± 0.30	0.611
LDL (mmol/L)	3.7 ± 0.6	3.6 ± 0.6	0.168
TG (mmol/L)	1.5 ± 0.7	1.6 ± 0.8	0.889
ALP (IU/L)	76 ± 21	72 ± 18	0.826
AST (IU/L)	36 ± 14	38 ± 13	0.381
ALT (IU/L)	35 ± 16	39 ± 22	0.611
GGT (IU/L)	33 ± 19	32 ± 19	0.712
hs-Crp (mg/L)	4.3 ± 7.1	4.0 ± 5.4	0.865
Apo B (g/L)	1.25 ± 0.20	1.25 ± 0.21	0.952
Lp(A) (mg/dL)	17 ± 14	16 ± 16	0.449
Serum creatinine (μmol/L)	86 ± 13	78 ± 11	0.008
Fasting glucose (mmol/L)	5.4 ± 0.8	6.1 ± 2.3	0.557
IFG ≥ 7.0 mmol/L	2 (4.7%)	7 (15.9%)	
Systolic (mmHg)	134 ± 18	131 ± 15	0.524
Diastolic (mmHg)	80 ± 9	80 ± 9	0.976
Tobacco, current users	5 (11.6%)	5 (11.4%)	
Mild NAFLD (score 3)	34 (79.1%)	35 (79.5%)	
Moderate NAFLD (score 4)	7 (16.3%)	8 (18.2%)	
Severe NAFLD (score 5-6)	2 (4.7%)	1 (2.3%)	

Baseline biochemical, anthropometric and clinical parameters of all patients enrolled in the study (*n* = 87). Data are expressed as mean value ± SD; *P* < 0.05 is considered significant.

Table 2*Abbreviations: BMI*  Body Mass Index, *TC*  Total Cholesterol, *HDL* High Density Lipoprotein, *LDL* Low Density Lipoprotein, *TG* Triglycerides, *ALP* Alkaline Phosphatase, *AST* aspartate transaminase, *ALT* alanine transaminase, *GGT* gamma-glutamyl transpeptidase, *hs-CRP* high-sensitivity C-reactive protein, *ApoB* apolipoprotein B, *LP(A)* lipoprotein A, *IFG* Impaired Fasting Glucose, *NAFLD* Nonalcoholic Fatty Liver Disease.

[*Wilcoxon rank test; Numbers’ 09, GraphPad*].

Conversion factor mmol/L to mg/dL for optimal normal values:

TC 5.2 mmol/L = 200 mg/dL;

HDL 1.55 mmol/L = 60 mg/dL;

LDL 2.6 mmol/L = 100 mg/dL;

TG 1.7 mmol/L = 151 mg/dL.

### Outcomes of USG hepatic evaluation

As for primary objective (Table 3), applying the intention to treat analysis to all the 87 initially randomised subjects, 43 in the tocotrienols group and 44 in the placebo group, a statistically significant advantage was seen for tocotrienols *versus* placebo for the normalisation of hepatic echogenic response (*P* = 0.039; Odds Ratio [OR] = 2.411; 95% Confidence Interval [CI] = 0.896-6.488; Number Needed to Treat [NNT] = 6).

**Table 3:** Intention to treat analysis of enrolled study subjects

	**Completion of the study**	
** Baseline (n = 87)**	**Normal echogenic response**	**Fatty liver**
Tocotrienols (n = 43)	15/43	28/43
	*34.9%*	*65.1%*
Placebo (n = 44)	8/44	36/44
	*18.2%*	*81.8%*
Tocotrienols *vs* Placebo	***P = 0.038***	

Drop-out subjects were computed as having an unchanged diagnosis of fatty liver from baseline.

[Helmert-Pearson’s Chi Square; Numbers’09, GraphPad].

The secondary objective per protocol analysis of the subjects that concluded the study, thus underwent the final USG examination, showed that 15 out of 30 patients (50.0%) in the tocotrienols group were found with normal echogenic response compared to only 8 out of 34 patients (23.5%) of the placebo group. Patients randomised to tocotrienols after 1 year of treatment presented a statistically significant normalisation of echogenic response compared to placebo (*P =* 0.014; OR = 3.250; 95% CI = 1.117-9.456; NNT = 3.8).

Details of the clinical evaluation of subjects and relative changes after 1 year of treatment are presented in Table 4.

**Table 4:** Per protocol analysis of ultrasound examination

**Tocotrienols group (n = 30)**	**Changes at completion of the study**			
**Baseline**	**Negative**	**Mild**	**Moderate**	**Severe**
Mild (n = 22)	13/22	9/22	0/22	0/22
*73.3%*	*59.1%*	*40.9%*	*0.0%*	*0.0%*
Moderate (n = 6)	2/6	4/6	0/6	0/6
*20.0%*	*33.3%*	*66.7%*	*0.0%*	*0.0%*
Severe (n = 2)	0/2	1/2	0/2	1/2
*6.7%*	*0.0%*	*50.0%*	*0.0%*	*50.0%*
TOTAL	15/30	14/30	0/30	1/30
	*50.0%*	*46.7%*	*0.0%*	*3.3%*
**Placebo group (n = 34)**	**Changes at completion of the study**			
**Baseline**	**Negative**	**Mild**	**Moderate**	**Severe**
Mild (n = 27)	8/27	17/27	2/27	0/27
*79.4%*	*29.6%*	*63.0*	*7.4%*	*0.0%*
Moderate (n = 6)	0/6	5/6	1/6	0/6
*17.6%*	*0.0%*	*83.3%*	*16.7%*	*0.0%*
Severe (n = 1)	0/1	0/1	1/1	0/1
*2.9%*	*0.0%*	*0.0%*	*100.0%*	*0.0%*
TOTAL	8/34	22/34	4/34	0/34
	*23.5%*	*64.7%*	*11.8%*	*0.0%*
Tocotrienols *vs* Placebo	***Chi Sq*** **= 4.851**	***P*** **= 0.014**		

Details of the clinical evaluation of subjects at baseline and relative changes after 1 year of treatment.

[Helmert-Pearson’s Chi Square; Numbers’09, GraphPad].

Worsening of steatotic grade was seen in two cases in the placebo group, but none in the tocotrienols group. Furthermore, amelioration of steatotic grade of more than one degree has been recorded in one male subject of the tocotrienols group, that improved from severe to mild and in two male subjects that improved from moderate to negative. Whereas no changes of more than one degree were observed in the placebo group.

### Blood biochemistry

No statistically significant changes were seen amongst the two groups of subjects between baseline and conclusion of the study (Table 5) with regard to blood biochemistry results. Exception was ApoB, that showed a statistically (*P* = 0.046), but not clinically significant difference since the values were within the normal range. This difference was explained with gender difference for normality range, with 0.63-1.33 g/L and 0.60-1.26 g/L for male and female respectively. Overall, blood biochemistry results indicated that tocotrienols did not significantly affect lipid profile and liver function when compared with placebo. However, a statistically significant reduction of total cholesterol (*P* = 0.008), LDL (*P* = 0.041) and TG (*P* = 0.027) in the tocotrienols group compared to baseline was observed. Within the placebo group TG values were significantly decreased compared to baseline in similar magnitude as the treatment group (*P* = 0.027). On the other hand, reduction of total cholesterol (*P* = 0.210) and LDL (*P* = 0.106) compared to baseline were not significant.

**Table 5:** Subjects parameters changes

**Variable**	**Tocotrienols (n = 30)**	**Placebo (n = 34)**	***P *Value (Tocotrienols vs Placebo)**
BMI (kg/m^2^)	-0.6	-0.3	0.527
TC (mmol/L)	-0.3	-0.2	0.055
HDL (mmol/L)	-0.04	0.02	0.778
LDL (mmol/L)	-0.2	-0.1	0.052
TG (mmol/L)	-0.2	-0.2	0.814
ALP (IU/L)	-7.0	1.4	0.471
AST (IU/L)	-4.8	-3.4	0.207
ALT (IU/L)	-5.9	-0.6	0.118
GGT (IU/L)	-1.4	-4.2	0.364
C-rp (mg/L)	-0.2	-0.2	0.835
Apo B (g/L)	-0.01	-0.03	0.046
Lp(A) (mg/dL)	2.3	0.4	0.524
Serum creatinine (μmol/L)	-5.1	-1.9	0.524
Fasting glucose (mmol/L)	0.5	0.5	0.490
Systolic (mmHg)	0.1	0.6	0.660
Diastolic (mmHg)	-0.3	-2.4	0.607

Mean changes in anthropometric and laboratory parameters for the study subjects after 1 year of treatment. *P* < 0.05 is considered significant.

[ANOVA for split-plot design adjusted for unweighted means; Numbers’09].

*Abbreviations as in* Table 2*.*

Plasma levels of alpha-tocopherol were in the normal reported range of 5.5-18.0 μg/ml [15] for all patients. The mean baseline levels of alpha-tocopherol were similar for the two groups of participants, being 13.0 ± 5.9 μg/ml and 14.6 ± 6.0 μg/ml for placebo and tocotrienols groups, respectively. At conclusion of the study, the values for alpha-tocopherol were substantially unchanged in the placebo group with a mean concentration of 13.4 ± 7.0 μg/ml. Whereas, in the tocotrienol group the values of alpha-tocopherol were found to be 18.5 ± 6.8 μg/ml. Nevertheless, the changes in alpha-tocopherol levels were found to be statistically non-significant (P = 0.665).

Baseline tocotrienol levels were negligible in both groups. Only alpha-tocotrienol was above the minimum limit of quantification, with 25.0 ± 21.0 ng/ml and 26.7 ± 32.6 ng/ml for tocotrienols and placebo group respectively. Gamma- and delta-tocotrienols were below the minimum limit of quantification in both groups.

High levels of tocotrienols were measured in all participating volunteers of the tocotrienols group at conclusion of the study, thus confirming a good level of compliance. Mean values were 212.0 ± 379.3 ng/ml, 181.4 ± 440.8 ng/ml and 41.4 ± 98.4 ng/ml for alpha-, gamma- and delta-tocotrienol respectively. On the other hand, they were negligible in the placebo group, with 21.7 ± 27.9 ng/ml for alpha-tocotrienol and below the minimum limit of quantification for gamma- and delta-tocotrienols.

## Discussion

Vitamin E, as alpha-tocopherol has been widely investigated in liver diseases earning the status of most promising treatment at a daily dosage of 533.3 mg (or 800 IU) in non-diabetics with biopsy-proven NASH [16]. The present study is the first to consider the hepatoprotective effects of tocotrienols. Their effectiveness in ameliorating NAFLD could in part be explained by the fact that the tocotrienols have been shown to distribute preferentially to the liver [6,7] where alpha-tocotrienol has been found to exert a 40-60 times more potent antioxidant activity in microsomal lipid peroxidation [8]. Oxidative stress has been recognised amongst the contributing factors in the progression of steatosis [2,17] with involvement of nuclear factor-κΒ (NF-κΒ) activation and increased release of proinflammatory cytokines, such as tumour necrosis factor alpha (TNFα) and the interleukin-6 (IL-6) representing the second insult in the “two hits” model [18,19]. Tocotrienols were reported to inhibit TNFα and to “prevent reactive oxygen species-induced NF-κΒ expression” to a higher extent compared to alpha-tocopherol in streptozotocin-treated diabetic rats [20]. Recently, biological mechanisms for the activity of tocotrienols against hepatic triglycerides accumulation have been reported. It has been shown that gamma-tocotrienol, but not alpha-tocopherol, was able to attenuate triglycerides accumulation by regulating fatty acid syntase and carnitine palmitoyltransferase leading to a reduction of hepatic inflammation and endoplasmic reticulum stress [21,22]. A 16-week study on rats fed high fat and high carbohydrate diet, showed a reduction of the hepatic fatty changes with “normalised portal inflammatory cells” [23]. The authors suggested that interference with the inflammatory processes to be one of the possible mechanisms for hepatoprotection [23].

Clinical trials employing the same mixed tocotrienols preparation used in the present study has been shown to be effective in improving arterial compliance [24] and in reducing Model for End-stage Liver Disease (MELD) score [7]. Moreover, the lipid lowering activity of tocotrienols has been previously reported [25] and a modest reduction of lipid profile was recorded also in the present study. Blood lipids biochemistry results showed that tocotrienols significantly reduced TC, LDL and TG compared to baseline, but not compared to placebo. However, the values for TC and LDL were still above the normality range.

In our study, baseline levels of alpha-tocopherol did not differ between the two groups. The plasma alpha-tocopherol concentrations of subjects in the tocotrienols treated group at conclusion of the study were found to be slightly higher than baseline, although not statistically significant. The presence of 61.1 mg of alpha-tocopherol in the mixed tocotrienols preparation administered may have contributed to slight levels increase of alpha-tocopherol. Nevertheless, mean plasma concentrations of alpha-tocopherol in the participating subjects were still within the normal range of 5.5-18.0 μg/ml reported by the National Reference Laboratory [15] throughout the study, irrespective of the treatment received. The presence of alpha-tocopherol in the mixed tocotrienols capsules was unlikely to have any influence on the outcomes of the present study since the amount present in the capsules was far below those used in earlier studies with negative outcomes [26-29].

We acknowledge that histology examination is the gold standard for evaluation of liver diseases and the use of USG might represent an intrinsic limitation. However, USG examination has been shown to have adequate sensitivity and specificity, in particular as front line evaluation of non-advanced fatty liver.

Diagnostic reports showed that USG has specificity range of 60-100% and sensitivity range of 80-100% [30-32]. Obesity with BMI above 40 might affect USG evaluation, reducing specificity and sensitivity when fat infiltration is below 33% [33]. In the present study, only one morbid obese was enrolled whose baseline diagnosis of NAFLD was unchanged at conclusion.

A gross advantage of USG is that the liver is seen in full, while a needle biopsy only samples about one 50,000^th^ of the organ and area sampling error could be an issue in non-advanced cases [31,34]. The choice of investigating hepatoprotective activity of a nutritional supplement, such as mixed tocotrienols, in adults drawn from a population-based study [10] with the use of non-invasive USG has to be seen in this light, being the majority of patients classified as mild steatosis with normal to slightly elevated liver aminotransferases, thus a non-advanced stage of NAFLD.

A possible weakness of our study may lie in the use of USG versus other non-ionising imaging methodologies such as proton magnetic resonance spectroscopy (^1^H MRS). ^1^H MRS is a powerful and objective tool for assessing fat fraction in the liver, even though it lacks of “information about regional fat distribution” [35].

A recent study [36] aimed at correlating ^1^H MRS with the echodiscrepancy ratio between kidney and liver as indicator of steatosis, on subjects with essentially similar anthropometric and blood biochemical values to our present study, concluded that the hepatorenal echodiscrepancy ratio evaluated with USG is in accord with the quantitative data obtained from ^1^H MRS for hepatic lipid infiltration in the range 1%-30% for comparable severity of steatosis, albeit what Mancini [36] considered as moderate in their study, is categorised as mild in ours.

We acknowledge that our patients were a low-risk cohort. However, patients with similar characteristics as those considered in our study, including normal liver transaminases levels, have been shown to still be at risk with respect to progression of NAFLD [37,38].

In view of the not fully elucidated reasons for steatosis to progress to more advanced stages of liver disease, it might be beneficial to manage the condition during the early stages, such as at the steatotic stage. Hence, in view of our findings, mixed tocotrienols might be considered for the treatment of steatosis.

In summary, the present study showed that patients assigned to tocotrienols at the current dosage, have significantly benefited in terms of normalisation of the hepatic echogenic response in NAFLD compared to placebo during the 1-year treatment. We acknowledge that this is a pilot study and therefore larger cohort studies should be warranted to further confirm the positive results of tocotrienols in NAFLD of the present study. Furthermore, at the current combined dosage of 400 mg/d used in the present study, the mixed tocotrienols were well tolerated and no adverse reactions were reported.

## Abbreviations

NAFLD: Non-alcoholic fatty liver disease; NASH: Non-alcoholic steatohepatitis; BMI: Body mass index; TC: Total cholesterol; HDL: High density lipoprotein; LDL: Low density lipoprotein; TG: Triglycerides; ALP: Alkaline phosphatase; AST: Aspartate transaminase; ALT: Alanine transaminase; GGT: Gamma-glutamyl transpeptidase; hs-CRP: High-sensitivity C-reactive protein; ApoB: Apolipoprotein B; LP(A): Lipoprotein A; IFG: Impaired fasting glucose; HPLC: High-performance liquid chromatography; USG: Ultrasonography; CI: Confidence interval; NNT: Number needed to treat; OR: Odds ratio; NF-κΒ: Nuclear factor-κΒ; TNFα: Tumour necrosis factor alpha; IL-6: Interleukin-6; 1H MRS: Proton magnetic resonance spectroscopy; HCC: Hepatocellular carcinoma.

## Competing interests

JW Wong, BH Ng, KH Yuen contributed a chapter (“Absorption and disposition of tocotrienols”) in the book: “Tocotrienols: vitamin E beyond tocopherols” Watson&Preedy Eds, CRC Press, Boca Raton, FL, USA. KH Yuen has patents related to the investigational product (self-microemulsifying delivery system for fat-soluble drugs: US patent 6596306). KH Yuen is an independent consultant for Hovid. KH Yuen, JW Wong, BH Ng & E Magosso own shares of Hovid. None of the authors is a substantial shareholder of Hovid. E Magosso, KH Yuen & IL Shuaib filed a patent application for the indication of tocotrienols in prevention of liver diseases. KH Yuen & E Magosso have served as speakers for Hovid, but without receiving any financial remuneration or gratuity. Y Gopalan, MA Ansari, NAK Khan, MR Abu Bakar have no potential conflict of interest.

## Authors’ contributions

Guarantors for the article: KHY & EM. ILS, MAA performed the Radiological Investigations and Clinical Evaluations; MRAB contributed to the Radiological Investigations and Clinical Evaluations. EM, KHY, ILS and NAKK designed the research protocol and wrote the manuscript; EM and YG collected and analysed the data; JWW was responsible for the randomisation; BHN contributed to the randomisation. All authors read and approved the final manuscript.
